# Toward a Better Understanding of Human Prion Diseases

**DOI:** 10.1371/journal.pmed.0030055

**Published:** 2005-12-20

Misfolding of a single protein, the cellular prion protein (PrP ^c^) into the disease-associated form PrP ^Sc^ is believed to cause fatal prion diseases in humans and other mammals, including sheep, cattle, and deer. The misfolding can occur sporadically or after contact—through inoculation or ingestion—with PrP ^Sc^ from an external source. There are also familial forms of prion diseases that are associated with certain mutations in *PNRP*, the gene encoding PrP ^c^. These abnormal PrP ^c^ proteins are thought to have a higher probability to misfold than normal PrP ^c^. The idea is that a few misfolded molecules can initiate a chain reaction and cause transformation of many of the other PrP ^c^ molecules into harmful PrP ^Sc^ versions. The presence of PrP ^Sc^ proteins causes widespread cell death, leading to the characteristic spongiform degeneration of the brain that kills patients, most of them within a matter of months.

The most common human prion disease is sporadic Creutzfeld –Jacob Disease (sCJD). The disease is rare (affecting roughly one to two individuals per a million people, worldwide), and its etiology is unclear; neither exogenous nor endogenous causes have been identified. sCJD is inevitably fatal, but the disease is clinically, pathologically, and genetically heterogeneous. Most patients have rapidly progressing dementia, often accompanied by involuntary muscle spasms, and death occurs within months of the first clinical symptoms. However, for some patients ataxia is the first clinical sign, while others develop sight problems, and for some the disease duration can be more than two years. In the hope that understanding the heterogeneity will help them to understand what causes sCJD, researchers are trying to systematically collect and catalog data from patients. To do this in a meaningful way, standardized assays that allow results from different patients and different laboratories to be compared in a meaningful way are necessary. [Fig pmed-0030055-g001]

**Figure pmed-0030055-g001:**
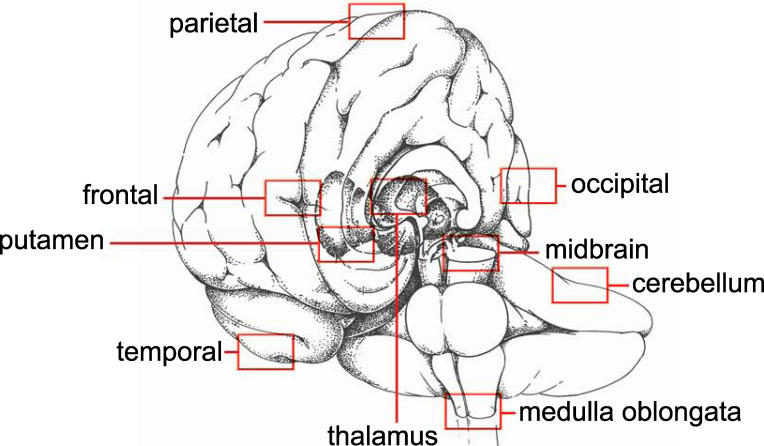
PrP ^Sc^ profiling was done in nine different central nervous system areas

Markus Glatzel and colleagues have developed such an assay, and applied it as part of the detailed molecular characterization to autopsy samples from 50 patients with sCJD. The new assay, which the researchers call PrP ^Sc^ profiling, measures the amount of PrP ^Sc^ in defined brain regions. In the past, PrP ^Sc^ amounts were routinely only measured in one or two regions by a variety of assays. PrP ^Sc^ profiling quantifies the amount of PrP ^Sc^ in nine defined brain regions relative to internal standards, and thereby allows for direct comparison of individual profiles.

The researchers determined PrP ^Sc^ profiles of 50 patients, and tried to correlate the profiles with information on disease types of the patients and prion types present in the different brain areas. sCJD types are determined by a patients' *PRNP* genotype at the polymorphic position 129 of *PRNP* and by the relative resistance of PrP ^Sc^ to proteolytic degradation. It is thought that most patients only have one prion type, but previous reports have described coexistence of two different types in some samples.

Analysis of this wealth of data revealed correlations between distinct PrP ^Sc^ distribution patterns and sCJD subtypes. These results have implications for confirmation of sCJD by brain biopsy. Before doing such biopsies, Glatzel and colleagues suggest, the sCJD subtype should be determined so that the correct brain area is examined. The researchers also found coexistence of two different prion types in 20% of their overall samples, and in more than 50% of the samples from patients who were heterozygous for the 129 polymorphism in the *PRNP* gene. These data lend further support to a link between molecular signature and clinical heterogeneity of the disease.

While many questions remain, this study underlines that the systematic analysis of prion cases can reveal links between molecular pathology, genetic makeup of patients, and disease symptoms. Glatzel and colleagues believe that “PrP ^Sc^ profiling will be a valuable tool for prion research.” In the hope that it will “facilitate comparisons of PrP ^Sc^ quantities present in defined samples,” the researchers will make their PrP ^Sc^ standard available to the scientific community.

